# QTL analysis of sorghum grain traits based on high-density genetic map

**DOI:** 10.1007/s13353-024-00904-w

**Published:** 2024-09-21

**Authors:** Ning Cao, Yanqing Ding, Jianxia Xu, Bin Cheng, Xu Gao, Wenzhen Li, Guihua Zou, Liyi Zhang

**Affiliations:** 1 Guizhou Institute of Upland Crops, Guizhou Academy of Agricultural Sciences, Guiyang, 550006 China; 2 Institute of Crop and Nuclear Technology Utilization, Zhejiang Key Laboratory of Digital Dry Land Crops, Zhejiang Academy of Agricultural Sciences, Hangzhou, 310021 China

**Keywords:** Candidate genes, Genetic mapping, Grain traits, Quantitative trait locus (QTL), Sorghum

## Abstract

Sorghum grain traits are important agronomic traits directly related to yield and are key factors affecting the brewing process of distill liquor. Exploring the genes controlling those traits is of great significance for understanding the genetic mechanism of sorghum grain development. In this study, we conducted genotyping using Super-GBS technology on a recombinant inbred lines (RILs) population derived from the cross between “BTx623” and “Hongyingzi,” consisting of 205 lines. The grain-related traits of the RIL population were investigated in Guiyang, Anshun in Guizhou, and Ledong in Hainan in China. By inclusive composite interval mapping (ICIM) method, a total of 47 quantitative trait locus (QTL) related to four grain traits (thousand grain weight, grain length, grain width, and length–width ratio) were identified across 10 chromosomes. Among them, 20 important QTL were repeatedly detected in multiple traits or environments and distributed on chromosomes 1 (1), 2 (2), 3 (5), 4 (5), 5 (1), 6 (2), 7 (2), 8 (1), and 9 (1). Six candidate genes were identified within the confidence interval of these QTL, and they are homologous to genes controlling rice grain development (*OsMADS1*, *RGG2*, *OsNST1*, *SMG1*, *OsGRF8*, and *OsAP2-39*). The results provide a basis for further cloning and functional verification of these candidate genes.

**Supplementary Information:**

The online version contains supplementary material available at 10.1007/s13353-024-00904-w.

## Introduction

Sorghum (*Sorghum bicolor* (L.)Moench, 2n = 2x = 20) is the world’s fifth-largest cereal crop and has a long history of cultivation in China (Ding et al. 2019; Morris et al. 2013; Lu 1999). It is recorded that sorghum has been utilized for brewing liquor in China since the Yuan Dynasty (1270–1368CE) (Shinoda 1958; Zhang et al. 2023a, b). Currently, sorghum is mainly grown in the southwest, north, and northeast regions of China, with over 80% of sorghum grains used to produce distill liquor (Chen et al. 2019; Li et al. 2021; Shinoda 1958; Tang and Xie 2019). In the southwest region, almost all sorghum grains are predominantly utilized for liquor brewing, with a historical emphasis on the production of renowned Maotai-flavored liquor. Sorghum grain traits are not only closely related to yield but also play a critical role in the brewing process (Gao et al. 2022), which comprises over 30 steps and requires small and round grains to obtain good resistance to cooking and stirring (Feng et al. 2022). The increasing scale of Maotai-flavored liquor production in recent years has led to a growing demand for sorghum cultivation. However, there are issues such as severe degradation and low yield in sorghum varieties for liquor, which affect the supply of raw materials for liquor production (Ding et al. 2020). Therefore, QTL mapping and gene cloning for grain related traits, as well as developing tightly linked molecular markers, are significant for improving the efficiency of breeding high-yielding sorghum varieties for liquor brewing purposes.

Grain-related traits are complex quantitative traits influenced by both genotype and environment (Baye et al. 2022; Tao et al. 2018). With the continuous improvement of sorghum genome, and the development of high-throughput sequencing technology, numerous QTL controlling grain related traits have been identified in sorghum (Han and Cai 2019). Currently, utilizing parental populations and natural populations, 29 studies have reported 186 QTLs affecting grain related traits on the 10 chromosomes of sorghum (aussorgm.org.au). Among these, grain weight has received the most attention as it is one of the three essential components contributing to yield, with over 80% of the QTLs related to thousand grain weight (TGW). However, there have been few reports on QTL for other grain related traits, with only 18 QTL detected for grain length (GL), 4 for grain width (GW), 4 for grain length–width ratio (GLWR), and 8 for grain roundness (GR). QTL for GL and GW are located on all chromosomes, except for chromosome 8, whereas QTL for GLWR and GR are found on chromosomes 1, 3, 6, 7 and 9 (Guindo et al. 2019; Liu et al. 2019; Zhang et al. 2015).

Despite numerous studies on QTL mapping of sorghum grain-related traits, there is a few report of fine mapping or gene cloning of QTL, which lags behind the research in major cereal crops such as rice, maize, and wheat (Boyles et al. 2016; Han et al. 2015; Tao et al. 2020; Zou et al. 2020). Han et al. finely mapped of a major QTL (*qGW1*) for GW within a range of approximately 101 kb on the short arm of chromosome 1, and *Sobic.001G038300* was preliminarily determined as the candidate gene for *qGW1* (Han et al. 2015). Boyles et al. discovered a novel QTL for TGW in the 5 M interval of chromosome 5, with the peak marker located on the gene *Sobic.005G188400* encoding an unknown function remorid protein, which is mainly expressed in early floral tissues and developing embryos (Boyles et al. 2016). Based on resequencing of a RIL population of 244 lines, Zuo et al. detected a major QTL (*qTGW1a*) on chromosome 1 and employed map-based cloning to identify *Sobic.001G341700* as the candidate gene, which is homologous to the gene *GS3* controlling grain size in rice. Functional analysis revealed that *qTGW1*a acts as a negative regulator of grain size in sorghum (Zou et al. 2020). Using genome-wide association analysis (GWAS) and nested association mapping, Tao et al. identified an important QTL (*qFHGS2.5*) on chromosome 2. They further determined the candidate gene (*Sobic.002G216600*) which is homologous to DEP1 in rice, a positive regulator of grain number related to grain weight (Tao et al. 2020).

To date, the sorghum accessions used for QTL mapping and GWAS analysis mainly originate from foreign resources (https://aussorgm.org.au/sorghum-qtlatlas/). However, significant differences exist between Chinese sorghum and foreign sorghum, and there may be new important QTL loci controlling grain traits. Therefore, in this study, utilizing the high-density linkage map, QTL related to sorghum grain traits was detected in the RIL constructed from Chinese and foreign sorghum (Ding et al. 2023), and candidate genes for the significant QTL were identified, which would provide a basis for further gene cloning, functional analysis, and molecular breeding of sorghum grain traits.

## Materials and methods

### Plant materials

A set of 205 RILs derived from a cross between sorghum inbred lines BTx623 and Hongyingzi (HYZ) was developed and used in this study. There were significant differences in panicle type and grain traits between parents. The Inbred line BTx623 had a compact panicle with large and round seeds, and HYZ had a large and loose panicle with small and oval seeds. Each RIL was derived from a signal F_2_ plant by single seed descent until the F_12_ generation.

In field trials, the 205 lines and their parents were planted for investigation of grain traits in Guiyang in 2020 (20GY) and 2021(21GY), Guizhou Province [26.67°N, 106.62°E, altitude 1175 m]; in 2021 in Anshun (21AS), Guizhou Province [25.21°N, 105.13°E, altitude 1280 m]; and in 2021 in Ledong (21HN), Hainan Province [18.74°N, 109.17°E, altitude 28 m]. Planting seasons in different environments (sowing in April and harvesting in August in Guizhou Province, sowing in November and harvesting in March the following year in Hainan Province) have the following total rainfall and average temperature for 20GY, 21GY, 21AS, and 21HN respectively: 933.94 mm/20.84 °C, 1089.44 mm/20.58 °C, 918.04 mm/22.35 °C, and 155.6 mm/19.59 °C (for specific information, please see Table S1). We fertilized 750 kg compound fertilizer (N + P2O5 + K2O ≥ 45%) per hectare in the field before planting Sorghum. The ratio of nitrogen, phosphorus, and potassium is 15–15-15 in the compound fertilizer.

### Phenotyping

Two hundreds grams of with intact and undamaged seeds were selected to measure the thousand grain weight (TGW), grain length (GL), grain width (GW), and length–width ratio (GLW), using the Wanshen SCG automatic seed analyzer (Hangzhou Wanshen Testing Technology Co., Ltd.). TGW is recorded in grams, and the measurements are considered valid if the difference between three weight measurements is less than 0.1 g (within 5%), and then the average value is then taken. GL and GW are measured in millimeters, with each line repeated three times, and the average value is calculated. The GLW is obtained by dividing GL by GW (for specific information, please see Table S2). Descriptive statistical analyses, including mean, standard deviation, and frequency distribution, as well as correlation analyses, were conducted on the grain traits across different environments using SPSS 21.0 software. Graphs were generated using R4.03 software.

### QTL mapping and candidate gene prediction

Based on the constructed genetic linkage map (Ding et al. 2023), QTL analysis was performed using the inclusive composite interval mapping (ICIM) method in QTL Ici-Mapping 4.2 software. The parameters used were a stepwise regression probability of *P* < 0.001, chromosome step length of 0.1 cM, and the logarithm of the odds (LOD) threshold of 2.5. QTL which were named according to the “QTL + trait + chromosome + order on the chromosome” principle. Previously reported sorghum QTL information was searched on the Sorghum QTL Atlas (http://aussorgm.org.au/sorghum-qtl-atlas/). Candidate genes in QTL interval were searched in the BTx623 reference genome, and their putative functions were annotated in the available databases: phytozome (https://phytozome-next.jgi.doe.gov/) and rice (https:// www. riced ata. cn/ gene/).

### RNA extraction and transcriptome expression analysis

Differences of samples were collected from the parents BTx623 and HYZ at four different stages of panicle/seed development (5 days after flowering in young panicles, 10 days after flowering in seeds, 15 days after flowering in seeds, 20 days after flowering in seeds). Total RNA was extracted from the samples. The NEBNextⓇ UltraTM RNA Library Prep Kit (New England BioLabs, USA) was used for transcriptome library construction, and the libraries that passed quality control were sequenced on the NovaSeq 6000 platform (Illumina, USA). After sequencing, the Filter-FQ software was used to remove adapters and low-quality sequences. The clean data were then aligned to the reference genome BTx623 using the HISAT2 software. The expression levels of genes were quantified using the cufflinks software, which calculated the gene FPKM (Fragments Per Kilobase of transcript per Million mapped reads) values. Three biological replicates were performed to calculate the mean ± SD expression levels for each sample.

## Results

### Phenotypic variation of the parents and RILs

From 2020 to 2021, the phenotype of the parents and RILs were surveyed in four environments, including Guiyang and Anshun, as well as Ledong (Table 1). The mean TGW, GL, GW, and GLWR of HYZ were 21.61 g, 4.11 mm, 3.35 mm, and 1.23, and the respective values of BTx623 were 24.77 g, 4.08 mm, 3.67 mm and 1.11. The distributions of four traits in the RIL population were continuous with coefficients of variation ranging from 0.04% to 0.54%, indicating the quantitative inheritance of these characteristics. The average values of these traits fell within the range of the two parental phenotypic values, and both the minimum and maximum values exceeded the parental phenotypic values. In addition, except for GLWR, the skewness and kurtosis absolute values of the RIL population’s TGW, GL, and GW were all less than 1, indicating that these traits followed a normal distribution.

**Table 1:** Phenotypic statistics of seed traits in parental and RIL populations across four environments

Trait	Environment	Parents		RILs						
		HYZ	BTx623	Range	Min	Max	Average	CV (%)	Skewness	Kurtosis
TGW (g)	2020GY	20.42	23.83	24.15	8.45	32.60	19.64	0.21	− 0.05	0.08
	2021AS	21.73	24.50	18.86	10.61	29.48	20.34	0.16	0.11	− 0.11
	2021GY	22.73	25.98	24.25	14.07	38.32	21.67	0.17	0.76	1.46
	2021HN	21.57	24.78	21.63	14.30	35.93	22.83	0.16	0.44	0.25
GL (mm)	2020GY	4.02	4.00	1.43	3.17	4.60	3.81	0.07	0.15	0.06
	2021AS	4.18	4.12	1.16	3.26	4.41	3.82	0.06	0.23	− 0.21
	2021GY	4.13	4.13	1.42	3.29	4.70	3.89	0.06	0.23	0.57
	2021HN	4.14	4.08	1.29	3.46	4.74	4.04	0.06	0.28	− 0.26
GW (mm)	2020GY	3.32	3.61	1.19	2.73	3.92	3.37	0.07	− 0.31	0.02
	2021AS	3.40	3.72	1.09	2.84	3.93	3.37	0.06	0.11	0.04
	2021GY	3.34	3.75	1.39	2.90	4.29	3.41	0.06	0.34	0.55
	2021HN	3.34	3.58	1.09	2.92	4.01	3.44	0.06	0.12	0.16
GLWR	2020GY	1.22	1.11	0.27	1.07	1.34	1.14	0.04	1.25	1.52
	2021AS	1.23	1.11	0.26	1.08	1.34	1.14	0.39	1.52	2.78
	2021GY	1.24	1.10	0.26	1.08	1.34	1.14	0.45	1.44	2.06
	2021HN	1.24	1.14	0.32	1.08	1.40	1.18	0.54	0.67	0.18

*TGW* thousand grain weight, *GL* grain length, *GW* grain width, *GLWR* grain length–width ratio, *2020GY* 2020 in Guiyang, *2021AS* 2021 in Anshun, *2021GY* 2021 in Guiyang, *2021HN* 2021 in Hainan. The same applies to the following

In order to understand the relationship between various traits under different environments, Pearson correlation analysis was conducted for four traits (Table 2). The results showed that TGW was significantly negatively correlated with GLWR in all four environments (*P* < 0.01), with a range of R values from − 0.18 to − 0.32. TGW was significantly positively correlated with GL and GW in all four environments (*P* < 0.01), with a range of R values from 0.69 to 0.91.

**Table 2:** Correlation analysis of Sorghum grain traits

Environment	Trait	TGW	GLWR	GL
2020GY	GLWR	− 0.18*		
	GL	0.79**	0.32**	
	GW	0.91**	− 0.27**	0.83**
2021AS	GLWR	− 0.27**		
	GL	0.74**	0.25**	
	GW	0.87**	− 0.39**	0.79**
2021GY	GLWR	− 0.32**		
	GL	0.71**	0.268**	
	GW	0.89**	− 0.436**	0.75**
2021HN	GLWR	− 0.22**		
	GL	0.69**	0.44**	
	GW	0.89**	− 0.41**	0.64**

^*^ and ** indicate significant correlation at the 0.05 and 0.01 levels, respectively

### QTL mapping for four grain traits

**Fig. 1:**
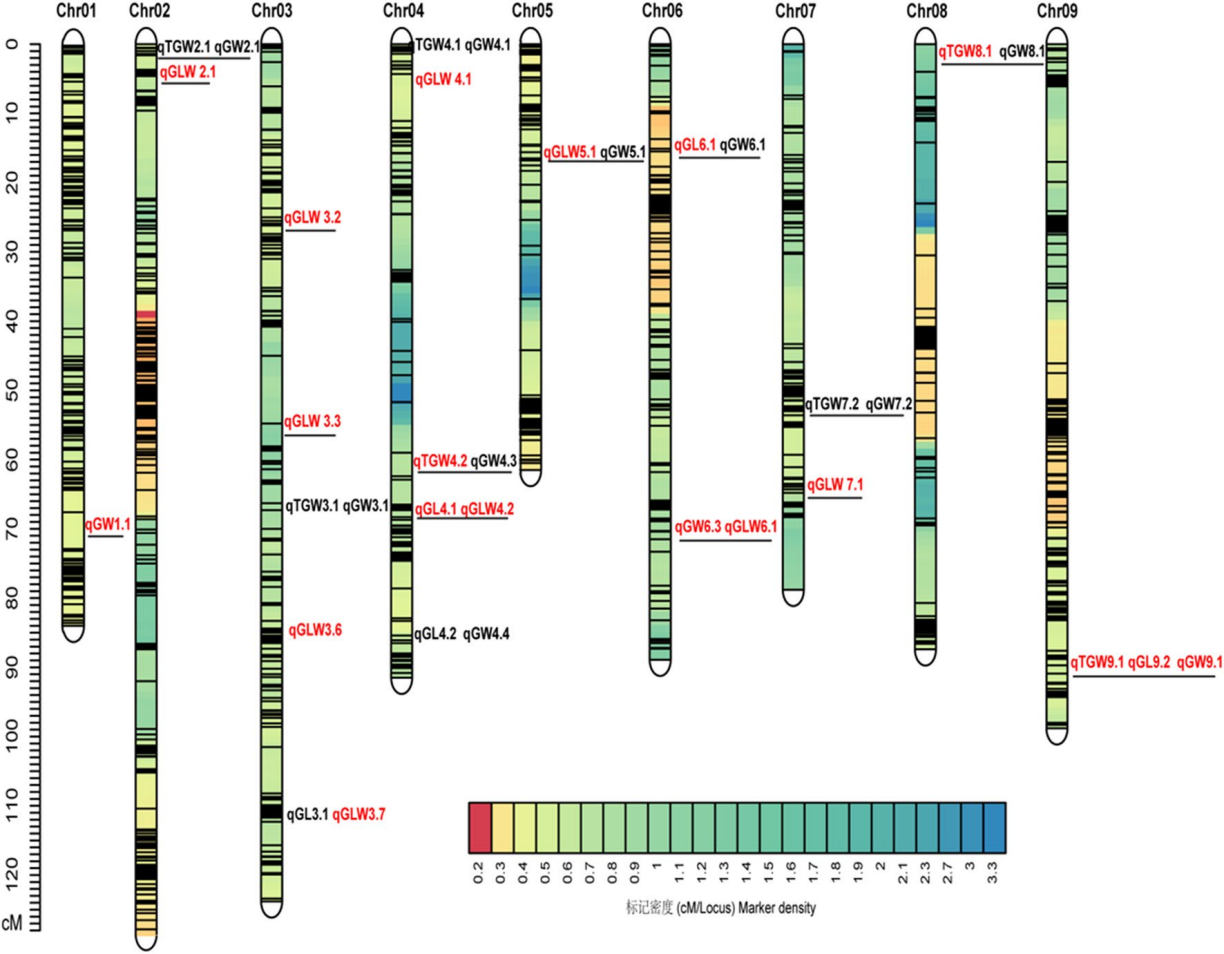
Schematic diagram of genetic map and the important QTL regions. QTL identified in single environment and in multiple environments are shown in black and red, respectively. Black underline indicates that QTL is consistent with previously reported QTL for sorghum grain traits

A total of 47 QTL related to the four grain traits were detected across all 10 chromosomes using the ICIM method across four environments (Table 3, Fig. 1). Of these, 8 QTLs were associated with TGW, 6 QTLs with GL, 15 QTLs with GW, and 18 QTLs with GLWR, involving a total of 35 unique loci.

**Table 3:** Summary of QTL mapping for four grain traits

Trait	Env	Chr	QTL	Pos. (cM)	Marker interval	Phy. (Mb)	LOD	PVE (%)	Add
TGW	21GY	2	*qTGW2.1*	6.70	sb2054–sb2502	1.88–1.87	4.63	6.23	0.96
	21GY	3	*qTGW3.1*	65.50	sb5560–sb5571	56.23–56.27	7.63	10.88	1.27
	21GY	4	*qTGW4.1*	0	sb6548–sb6572	1.30–1.48	3.29	4.37	0.81
	21HN		*qTGW4.2*	60.90	sb7891–sb7937	55.91–57.11	2.50	4.67	0.83
	21AS			62.90	sb7938–sb7997	57.14–58.31	3.60	5.98	0.83
	21GY			62.90	sb7938–sb7997	57.14–58.31	6.83	9.45	1.18
	21GY	7	*qTGW7.1*	47.30	sb12446–sb12455	61.02–61.08	2.88	3.80	− 0.76
	21HN		*qTGW7.2*	53.80	sb12544–sb12554	62.78–62.92	5.14	10.04	− 1.22
	21GY	8	*qTGW8.1*	0	sb12781–sb12813	1.84–2.18	4.02	5.37	0.90
	21AS			0.80	sb12781–sb12813	1.84–2.18	4.90	8.79	1.03
	21HN			1	sb12781–sb12813	1.84–2.18	4.61	9.57	1.18
	20GY			5.20	sb12813–sb12820	2.18–2.48	3.25	6.05	1.07
	20GY	9	*qTGW9.1*	93.70	sb15384–sb15373	57.85–57.67	8.23	15.26	− 1.69
	21AS			93.70	sb15384–sb15373	57.85–57.67	7.46	13.06	− 1.23
GL	21HN	3	*qGL3.1*	115.30	sb6322–sb6370	72.18–72.55	3.41	5.15	− 0.06
	21HN	4	*qGL4.1*	66.80	sb8006–sb8004	58.59–58.59	6.19	9.34	0.09
	21GY			66.99	sb8004–sb8003	58.59–58.59	5.82	11.17	0.08
	20GY			67.40	sb8009–sb8028	58.59–58.59	3.18	6.41	0.07
	21AS			68.70	sb8028–sb8036	59.35–59.49	6.88	13.01	0.07
	21AS		*qGL4.2*	87.20	sb8314–sb8356	65.75–66.54	4.17	7.85	0.06
	21GY	6	*qGL6.1*	17.70	sb9747–sb9761	3.04–3.24	3.51	6.56	0.06
	21HN			17.70	sb9747–sb9761	3.04–3.24	6.52	9.88	0.09
	21AS			18.20	sb9747–sb9761	3.04–3.24	3.70	7.02	0.05
	21HN	9	*qGL9.1*	25.20	sb14354–sb14353	3.94–3.94	2.67	3.87	0.06
	20GY		*qGL9.2*	93.70	sb15384–sb15373	57.85–57.67	4.06	8.34	− 0.08
	21AS			93.70	sb15384–sb15373	57.85–57.67	3.22	5.89	− 0.05
GW	21GY	1	*qGW1.1*	70.10	sb1847–sb1967	66.30–71.55	2.50	3.08	− 0.04
	21AS			73.40	sb1964–sb2044	71.51–73.61	3	4.26	− 0.04
	21GY	2	*qGW2.1*	6.70	sb2054–sb2502	1.88–1.87	6.5	7.14	0.06
	21GY	3	*qGW3.1*	65.50	sb5560–sb5571	56.23–56.27	6.63	7.39	0.06
	21GY	4	*qGW4.1*	0.40	sb6548–sb6572	1.30–1.48	4.31	4.59	0.05
	21AS		*qGW4.2*	22.70	sb6842–sb6864	72.67–75.87	5.35	6.44	0.06
	21GY		*qGW4.3*	61.60	sb7891–sb7937	55.91–57.11	5.43	6.24	0.06
	21AS		*qGW4.4*	88.40	sb8347–sb8353	66.52–66.53	8.34	10.39	0.07
	21GY	5	*qGW5.1*	31.00	sb9247–sb9299	63.53–65.02	4.40	4.70	0.05
	21GY	6	*qGW6.1*	17.90	sb9747–sb9761	3.04–3.24	5.89	6.60	0.06
	21HN		*qGW6.2*	45.50	sb10542–sb10567	46.12–47.24	3.76	6.41	0.06
	21GY		*qGW6.3*	73.50	sb10981–sb11205	54.59–57.26	3.32	3.57	− 0.04
	21HN			76.50	sb10981–sb11205	54.59–57.26	4.56	8.29	− 0.07
	21AS			78.30	sb11205–sb11232	54.59–57.26	4.10	4.88	− 0.05
	21AS	7	*qGW7.1*	20.80	sb11522–sb11487	2.81–2.50	3.01	3.64	0.04
	21HN		*qGW7.2*	53.60	sb12544–sb12554	62.78–62.92	3.06	5.19	− 0.05
	21HN	8	*qGW8.1*	0.80	sb12781–sb12813	1.84–2.18	2.52	4.55	0.05
	20GY	9	*qGW9.1*	93.70	sb15384–sb15373	57.85–57.67	7.81	16.35	− 0.09
	21AS			93.70	sb15384–sb15373	57.85–57.67	10.06	12.93	− 0.08
	21GY			93.70	sb15384–sb15373	57.85–57.67	3.57	3.82	− 0.05
GLW	21HN	1	*qGLW1.1*	61.70	sb1766–sb1767	64.28–64.29	5.40	4.03	0.01
	20GY	2	*qGLW2.1*	7.60	sb2509–sb2505	1.95–1.93	7.37	8.31	− 0.01
	21GY			7.90	sb2508–sb2511	1.94–2.04	5.70	9.08	− 0.02
	21HN			8.00	sb2511–sb2512	2.04–2.04	12.99	10.68	− 0.02
	21HN	3	*qGLW3.1*	5.30	sb4755–sb4754	1.69–1.69	4.57	3.39	0.01
	20GY		*qGLW3.2*	27.40	sb5036–sb5055	7.77–7.93	3.46	3.73	− 0.01
	21HN			27.40	sb5036–sb5055	7.77–7.93	4.85	3.64	− 0.01
	20GY		*qGLW3.3*	56.40	sb5420–sb5426	51.81–52.69	5.97	6.58	− 0.01
	21HN			56.50	sb5426–sb5466	52.69–53.57	3.25	2.38	− 0.01
	21AS			56.60	sb5426–sb5466	52.69–53.57	5.98	7.94	− 0.01
	21GY		*qGLW3.4*	60.30	sb5520–sb5517	55.06–55.03	7.48	12.16	− 0.02
	21HN		*qGLW3.5*	84.60	sb5811–sb5832	61.71–62.00	8.44	6.56	0.02
	21GY		*qGLW3.6*	86.99	sb5839–sb5845	62.35–63.30	4.66	7.45	0.01
	20GY			87.30	sb5839–sb5845	62.35–63.30	3.50	3.73	0.01
	20GY		*qGLW3.7*	114.60	sb6322–sb6370	72.18–72.55	5.93	6.52	− 0.01
	21HN			114.60	sb6322–sb6370	72.1–72.55	3.73	2.75	− 0.01
	20GY	4	*qGLW4.1*	9.50	sb6631–sb6754	2.83–4.85	4.36	5.24	− 0.01
	21AS			11	sb6631–sb6754	2.83–4.85	2.64	3.35	− 0.01
	20GY		*qGLW4.2*	67.40	sb8009–sb8028	58.64–59.35	4.31	4.66	0.01
	21HN			68.20	sb8009–sb8028	58.64–59.35	7.19	5.51	0.02
	21AS	5	*qGLW5.1*	17.30	sb9163–sb9247	61.54–63.53	4.23	5.45	− 0.01
	21HN			18	sb9163–sb9247	61.54–63.53	7.79	6.27	− 0.02
	21HN	6	*qGLW6.1*	73.60	sb10981–sb11205	54.59–57.26	9.99	8.08	0.02
	21AS			74.60	sb10981–sb11205	54.59–57.26	2.74	3.84	0.01
	21HN	7	*qGLW7.1*	68.20	sb12262–sb12261	57.42–57.39	5.64	4.24	0.02
	20GY			78.80	sb12263–sb12235	57.43–56.27	5.27	5.75	0.01
	21HN	8	*qGLW8.1*	54.80	sb13724–sb13730	54.9–55.14	5.21	4.06	− 0.01
	20GY		*qGLW8.2*	78.70	sb13812–sb13988	58.25–60.76	3.31	4.36	0.01
	20GY	9	*qGLW9.1*	97.90	sb15435–sb15437	58.88–58.96	2.77	2.94	0.01
	21GY	10	*qGLW10.1*	32.90	sb16279–sb16269	55.07–54.66	3.17	4.90	0.01

Positive and negative additive effects refer to alleles from parents BTx623 and Hongyingzi, respectively

Eight QTLs for TGW were located on chromosomes 2 (1), 3 (1), 4 (2), 7 (2), 8 (1), and 9 (1), respectively. The *qTGW8.1* on chromosome 8 was detected in all four environments, with a maximum LOD score of 4.90 and a phenotypic contribution rate of 8.79%. An important QTL on chromosome 4 was detected in three environments, with a maximum LOD score of 6.83 and a phenotypic contribution rate of 9.45%. Except for *qTGW7.1*, *qTGW7.2*, and *qTGW9.1*, the rest of 5 QTLs with additive alleles from the parental line BTx623.

Six QTLs for GL were located on chromosomes 3 (1), 4 (2), 6 (1), and 9 (2), respectively. The *qGL4.1* on chromosome 4 was detected in all four environments, with an average LOD score of 5.52 and a range of 3.18 ~ 6.88, explaining an average phenotypic variation of 9.98% and a range of 6.41 ~ 13.01%. One QTL on chromosome 6 was detected in three environments, with a maximum LOD score of 6.52 and a phenotypic contribution rate of 9.88%. Except for *qGL3.1* and *qGL9.2*, the additive alleles of the other four QTLs originated from the parental line BTx623.

Fifteen QTLs for GW were located on chromosomes 1 (1), 2 (1), 3 (1), 4 (4), 5 (1), 6 (3), 7 (2), 8 (1), and 9 (1), respectively. Two important QTLs (*qGW6.3* and *qGW9.1*) detected in three environments were located on chromosome 6 and chromosome 9, with LOD score ranges of 3.32 ~ 4.56 and 3.57 ~ 10.06, and explained phenotypic variation ranges of 3.57 ~ 8.29% and 3.82 ~ 16.35%, respectively. Five QTLs (*qGW1.1*, *qGW1.2*, *qGW6.3*, *qGW7.2*, and *qGW9.1*) had additive alleles from HYZ, while the other 10 QTLs had additive alleles from BTx623.

Eighteen QTLs for GLWR were located on chromosomes 1 (1), 2 (1), 3 (7), 4 (2), 5 (1), 6 (1), 7 (1), 8 (2), 9 (1), and 10 (1). On chromosome 3, two QTLs (*qGLW2.1* and *qGLW3.3*) were detected in two environments, with LOD score ranges of 5.70 ~ 12.99 and 3.25 ~ 5.98, and explained phenotypic variation ranges of 8.31 ~ 10.68% and 2.38 ~ 7.94%, respectively. Apart from *qGLW2.1*, *qGLW2.2*, *qGLW3.2*, *qGLW3.3*, *qGLW3.4*, *qGLW3.7*, *qGLW4.1*, *qGLW5.1*, and *qGLW8.1*, with additive alleles from the parental line HYZ, the additive alleles for the other 9 QTLs originated from BTx623.

In summary, among the 35 unique QTLs associated with the four grain traits, 20 loci were repeatedly detected in multienvironments or multitraits (≥ 2) (Fig. 1). The three loci with the most clustered QTLs were located on chromosomes 4, 6, and 9, respectively. The six QTLs for GL and GLWR consistently colocalized on the 58.59 ~ 59.35 Mb region of chromosome 4. The five QTLs for GW and GLWR overlapped on the 54.59 ~ 57.26 Mb region of chromosome 6. The seven QTLs for GL, GW, and TGW overlapped on the 57.67 ~ 57.85 Mb region of chromosome 9.

### Candidate genes predicting and expression analysis

To identify candidate genes for 20 important QTLs detected in multienvironments or multitraits, we examined the genomic regions covered by each QTL in the reference BTx623 genome version on *Phytozome 13*. Among the 20 major QTLs, 6 candidate genes were identified from 6 of these QTLs. The six genes *Sobic.001G455900*, *Sobic.004G035200*, *Sobic.004G214100*, *Sobic.004G323600*, *Sobic.005G150900*, and *Sobic.006G210300* correspond to the rice homologous genes *OsMADS1*, *RGG2*, *OsNST1*, *OsMKK4*, *OsGRF8*, and *OsAP2-39,* respectively (Table 4).

**Table 4:** Functional annotation of candidate genes in QTL mapping

QTL	Gene	Physical position	Rice homologous gene	Proteins annotation	KEGG annotation	GO annotation
*qGW1.1*	*Sobic.001G455900*	Chr01: 73188105–73199446	*OsMADS1;GW3p6 LOC_Os03g11614*	MADS-box transcription	K09264	GO:0005634
*qGLW4.1*	*Sobic.004G035200*	Chr04: 2837130–2841378	*RGG2* *LOC_Os02g04520*	Guanine nucleotide-binding protein subunit gamma 2	K24772	GO:0005886
*qTGW4.2/qGW4.3*	*Sobic.004G214100*	Chr04: 56389819–56395087	*OsNST1; BC14* *LOC_Os02g40030*	Nucleotide-sugar uncharacterized transporter 3	K15281	GO:0000139
*qGL4.2/qGW4.4*	*Sobic.004G323600*	Chr04: 65819835–65821680	*OsMKK4; SMG1* *LOC_Os02g54600*	Mitogen-activated protein kinase 5	K13413	GO:0005737
*qGLW5.1/qGW5.1*	*Sobic.005G150900*	Chr05: 61984980–61988110	*OsGRF8* *LOC_Os11g35030*	Growth-regulating factor 8-like isoform X1		GO:0005634
*qGW6.3/qGLW6.1*	*Sobic.006G210300*	Chr06: 55996537–55997629	*OsAP2-39* *LOC_Os04g52090*	Ethylene-responsive transcription factor 4	K09286	GO:0005634

*S1* young panicle 5 days after flowering, *S2* seeds 10 days after flowering, *S3* seeds 15 days after flowering, *S4* seeds 20 days after flowering

**Fig. 2:**
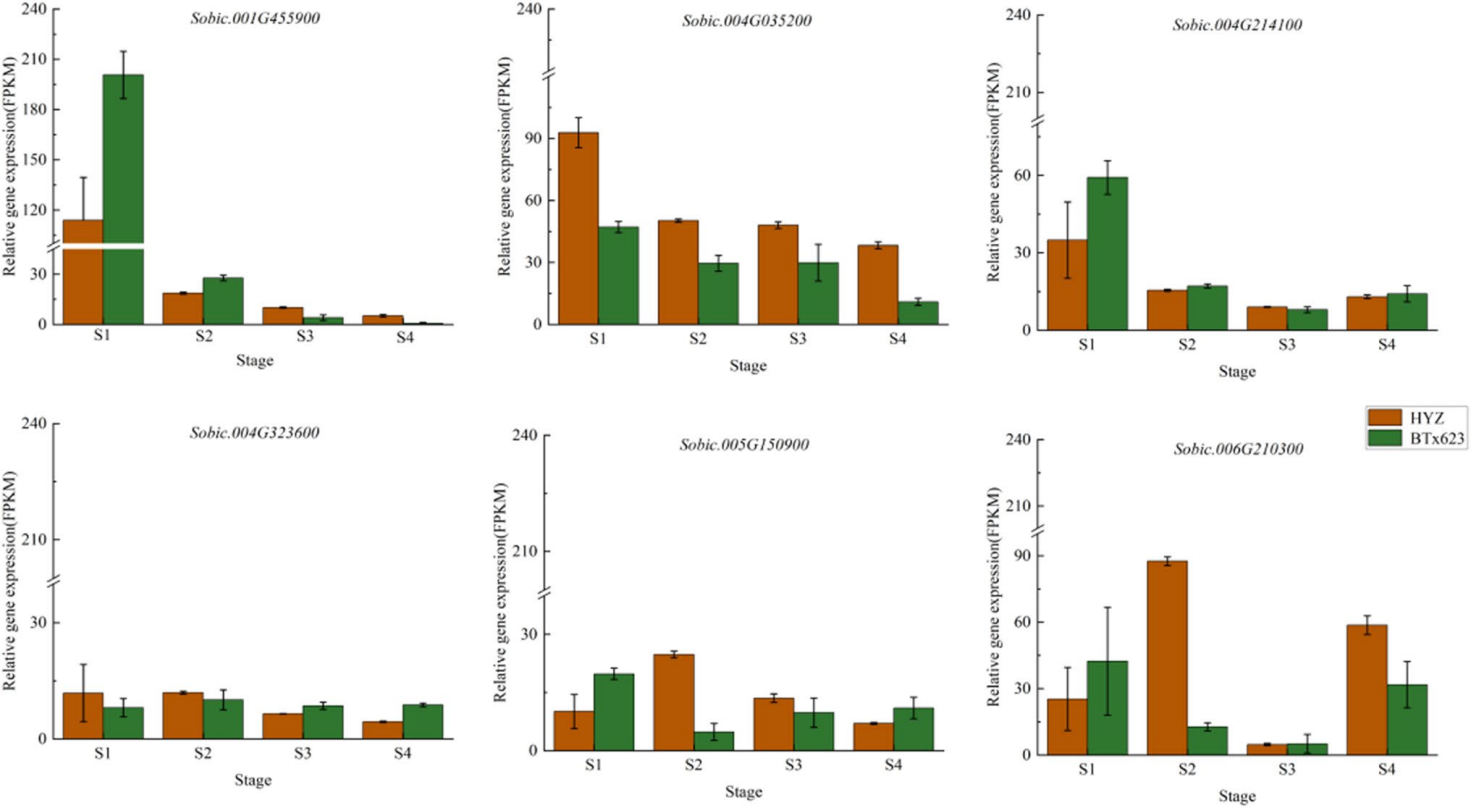
Transcriptome sequencing was used to analyze the expression patterns of candidate genes in HYZ (small grain) and BTx623 (large grain)

The expression levels of the six candidate genes showed significant differences in the four seed developmental stages between the parents (Fig. 2, Table S3). The expression trends of *Sobic.001G455900* and *Sobic.004G035200* were consistent in both parents, with expression levels gradually decreasing as panicle development progressed. For *Sobic.004G035200*, the expression levels in HYZ were higher than those in BTx623 across all four stages, with expression levels in the first and fourth stages being more than twice those in BTx623. The expression trend of *Sobic.004G214100* was similar in both parents, decreasing from S1 to S3 and slightly increasing at S4, with the highest expression levels occurring at S1. In the first two stages of HYZ, the *Sobic.004G323600* gene showed almost no change in expression levels gradually decreasing from the third stage, while expression levels in BTx623 remained stable across all four stages. The expression trends of *Sobic.005G150900* were completely opposite in the two varieties, with HYZ showing an increase followed by a decrease, reaching the highest level at S2, whereas BTx623 showed a decrease followed by an increase, reaching the lowest level at S2, with HYZ expression levels being more than four times those of BTx623 at this stage. *Sobic.006G210300* showed a high-high-low–high expression trend across the four stages in HYZ, whereas in BTx623, the trend was high-low-low–high, with both parents showing the lowest expression levels at S3 stage.

## Discussion

Since its introduction to China during the Yuan Dynasty, Sorghum has been an important ingredient for brewing famous distill liquor (Ding et al. 2023). In recent years, there has been a rapid increase in demand for the Maotai-flavor liquor, leading to a continuously expanding supply demand gap for brewing sorghum (Zhang et al. 2023a, b). Sorghum grain traits play a vital role in determining yield and brewing process. The utilization of molecular markers in modern breeding offers a unique opportunity to investigate the genetic control of numerous target traits and monitor the process of incorporating favorable alleles of genes involved in their variation. This study conducted QTL mining for grain-related traits, aiming to accelerate the breeding of high-yield sorghum varieties that meet the requirements of Maotai-flavor liquor production.

### QTL mapping for grain-related traits

Currently, extensive research has been conducted on grain-related traits in sorghum, and more than 180 relevant QTLs have been identified (Han and Cai 2019). Using the RIL population of BTx623/HYZ, this study repeatedly detected 20 important QTLs that affect grain size and shape in multi-environments or multi-traits, of which 14 loci are consistent with the reported QTL in previous research.

The QTL on chromosome 1 (*qGW1.1*, 66.30–73.61 Mb) overlaps with reported grain weight QTLs using four genetic mapping populations, BTx623/S.virgatum (Liu et al. 2019), CK60/China17 (Gelli et al. 2016), CK60/PI229828 (Pereira et al. 1995), and HYP/DTP (Phuong and Stützel 2013). Two adjacent QTLs on chromosome 2 (*qGW2.1/qTGW2.1* and *qGLW2.1*) overlap with the positions of grain roundness QTL reported using the RIL population P118 (Guindo et al. 2019). Two QTLs for GLWR were identified on chromosome 3. *qGLW3.2* is consistent with GL QTL using the RILs of P118 and grain weight QTL using the SA2313/Hiro-1 population (Han et al. 2015), while *qGLW3.3* is consistent with grain weight QTL mapping with the M35-1/B35 population (Nagaraja et al. 2013). On chromosome 4, two important QTLs (*qTGW4.2/qGL4.3* and *qGLW4.2/qGL4.1*) overlap with grain weight QTL detected using the R931945-2–2*2 / S.bicolor subsp.verticilliflorum population (Tao et al. 2018). The QTL on chromosome 5 (*qGLW5.1/qGW5.1*) is consistent with grain weight QTL obtained using the Red Kafir/ Takakibi (F2:3) population (Shehzad and Okuno 2015). The QTL on chromosome 6 (*qGLW6.1/qGW6.3*, 54.59–57.26 Mb) corresponds to the GW QTL obtained from the E-Tian/Ji2731 population (Mocoeur et al. 2015), while another QTL (*qGL6.1*) on chromosome 6 is consistent with grain weight QTL identified using GWAS analysis (Zhang et al. 2015). There are 2 QTLs on chromosome 7, with *qGLW7.1* overlapping with the position of grain weight QTL reported in the P114 population, and *qTGW7.2/qGW7.2* overlapping with grain weight QTL positions identified in two RIL populations (BTx623/ S.propinquum and SA2313/ Hiro-1) (Feltus et al. 2006; Han et al. 2015). The QTL affecting TGW and GW (*qTGW8.1/qGW8.1*) on chromosome 8 has the same position as grain weight QTL identified in the RIL population BTx6 (Boyles et al. 2017). On chromosome 9, the QTL (*qTGW9.1/qGL9.2/qGW9.1*) is consistent with grain weight QTL reported in the M35-1/B35 population (Nagaraja et al. 2013).

However, there are 6 important QTLs in this study (*qTGW3.1/qGW3.1*, *qTGW3.7/qGL3.1*, *qGL4.2/qGW4.4*, *qTGW4.1/qGW4.1*, *qGLW4.1*, and *qGL4.2/qGW4.4*) that have not been reported in pervious sorghum studies, which may be due to two reasons: Firstly, it could be influenced by differences in the genetic background, population type and size, marker type, and other factors of the mapping population. Currently, the parents of mapping populations for grain trait QTL mapping mostly come from American sweet cultivar, while one of the parents in this study is Chinese brewing glutinous variety “HYZ” and may carry different genes related to grain traits, resulting in differences in the number and position of QTLs (Tao et al. 2021). Additionally, this study used the Sup-GBS technology to construct a high-density map, enabling more precise mapping of QTLs for grain traits. Most of previous QTLs mapping on sorghum have used SSR markers (Han and Cai 2019).

### Candidate genes for grain-related traits

Among 20 important QTLs, 6 QTLs were identified candidate genes within its confidence interval. Which four QTLs have been reported in previous studies. The gene *Sobic.001G455900* in the interval of *qGW1.1* is homologous to *OsMADS1* in rice, which encodes a MADS domain transcription factor that regulates grain size and shape, improving the quality and yield of rice (Hu et al. 2015; Liu et al. 2018). The gene *Sobic.004G214100* within the *qTGW4.2/qGL4.3* interval is homologous to the BC14 gene in rice, which encodes a nucleotide sugar transporter in the Golgi apparatus, regulating the biosynthesis of seed cell walls and plant growth with pleiotropic effects (Song et al. 2011; Zhang et al. 2011). The gene *Sobic.005G150900* within *qGLW5.1/qGW5.1* interval is homologous to *OsGRF8*, a growth-regulating factor in rice by regulating flavonoid biosynthesis pathway, which increases rice yield by regulating GL and thousand grain weight (Dai et al. 2019; Yang et al. 2021). The gene *Sobic.006G210300* in *qGLW6.1/qGW6.3* is homologous to *OsAP2-39* in rice, which controls rice growth and seed production through the ABA/GA balance mechanism (Wan et al. 2011; Yaish et al. 2010).

Although two QTLs on chromosome 4 have not been previously reported, the gene *Sobic.004G035200* within the (*qGLW4.1*) interval is homologous to *RGG2*, which negatively regulates grain size, organ size, and yield of rice by participating in gibberellin biosynthesis (Miao et al. 2019). Another gene (*Sobic.004G323600*) in the interval of *qGL4.2/qGW4.4* is homologous to *OsMKK4/SMG1*, a key pleiotropic gene controlling rice yield, which regulates cell proliferation through the serine/threonine protein kinase *OsMKK4*, affecting grain size and environmental stress resistance in rice (Duan et al. 2014; Guo et al. 2020).

Currently, the study of functional genes influencing grain-related traits in sorghum lags significantly behind major cereal crops such as rice (Zhu et al. 2023), maize (Feng et al. 2023), and wheat (Zhang et al. 2023a, b). Only two genes have been cloned and functionally verified in sorghum until now (Tao et al. 2021; Zou et al. 2020). In order to further identify potential candidate genes in this study, subsequent analyses will include sequence divergence analysis among parents to determine genetic polymorphism, development of functional markers, and functional validation through gene editing techniques.

## Conclusions

Based on the genetic map, this study identified 6 QTLs for GL, 15 for GW, 8 for TGW, and 18 for GLWR across four environments. In addition, 20 significant QTLs were detected in multi-environments or multi-traits. Among them, six candidate genes potentially related to grain traits were identified, which are homologous to genes controlling grain size in rice. Our findings are critical to selecting target QTL/genes for improving grain-related traits in Chinese brewing sorghum in breeding program.

## Supplementary Information

Below is the link to the electronic supplementary material.

Supplementary file1 (XLSX 44 KB)

## Data Availability

The resequencing data generated in this study can be found in online repositories. The name of the repository and accession number can be found below: China National GeneBank (CNGB); CNP0005779. The website links are as follows: https://db.cngb.org/search/project/CNP0005779/.
